# Regulation of *Nicotiana benthamiana* cell death induced by citrus chlorotic dwarf-associated virus-RepA protein by WRKY 1

**DOI:** 10.3389/fpls.2023.1164416

**Published:** 2023-04-25

**Authors:** Yangyang Qin, Jinfa Zhao, Jiajun Wang, Xiao Ye, Changyong Zhou, Yan Zhou

**Affiliations:** National Citrus Engineering Research Center, Citrus Research Institute, Southwest University, Chongqing, China

**Keywords:** citrus chlorotic dwarf-associated virus (CCDaV), RepA, cell death, geminivirus, WRKY

## Abstract

Citrus chlorotic dwarf-associated virus (CCDaV) is a *Citlodavirus* species in the Geminiviridae family that causes tremendous economic loss to the citrus industry in China. Some proteins encoded by geminiviruses are crucial for the interaction between the virus and its host plant. However, the exact functions of CCDaV-encoded proteins such as CCDaV-RepA have not been investigated. This study presents evidence that CCDaV-RepA elicits a hypersensitive response (HR)-like cell death in *Nicotiana benthamiana* that was accompanied by the production of H_2_O_2_ and ion leakage, which suggested that CCDaV-RepA is a potential recognition target for inducing host defense responses. Furthermore, the rolling-circle replication motifs of CCDaV-RepA are associated with triggering HR-like cell death in *N. benthamiana*. Confocal microscopy and deletion mutagenesis assays showed that CCDaV-RepA was located in the nucleus, while the first eight amino acids (aa) at the N terminus and two regions located between aa residues 122-263 and 220-264 of RepA were not associated with nuclear localization. Tobacco rattle virus-induced gene silencing of the key signaling cascade components revealed that HR-like cell death induced by RepA was inhibited in *WRKY1*-silenced *N. benthamiana.* Moreover, *WRKY1* expression was upregulated in RepA-GFP infiltrated Overall, the results suggest that NbWRKY1 positively regulated CCDaV-RepA -induced cell death in *N. benthamiana*. These findings provide novel information for further research on the interactions between CCDaV and the host plant.

## 1 Introduction

Plants are particularly susceptible to infection by some viral diseases that can cause enormous economic loss by reducing yield and quality (Macho & Zipfel, 2014). Plants rely on several sophisticated antiviral immune responses for their survival and reproduction. The innate plant immune response is initiated when a plant-encoded *resistance* (*R*) protein is recognized by pathogens that carry *avirulence* (*avr*) genes, such as viral movement or coat proteins (Martin et al., 2003; Park & Kim, 2012). This recognition often leads to a hypersensitive response (HR) that involves rapid programmed cell death at the site of invasion (Zhou & Zhang, 2020). The HR is characterized by the release of reactive oxygen species (ROS) and nitric oxide (NO), ion fluxes, protein phosphorylation, the accumulation of signaling molecules such as salicylic acid (SA), ethylene (ET), and jasmonic acid (JA) and the activation of multiple defense genes, including pathogenesis-related genes (Mur et al., 2008).

Geminiviruses have one or two circular single-stranded DNA genomes in twinned icosahedral particles and are transmitted by a variety of insects, which can result in devastating crop losses worldwide (Navas-Castillo et al., 2011). According to their inferred genome organization, insect vectors, and host ranges, geminiviruses are classified into 14 genera (Roumagnac et al., 2022). Replication associated protein A (RepA) is unique to some geminivirus genera, including *Citlodavirus*, *Becurtovirus*, *Capulavirus*, *Mulcrilevirus*, *Grablovirus*, *Mastrevirus*, and *Topilevirus* (Sun et al., 2022). RepA is a multifunctional protein responsible for the transactivation of virus-sense open reading frames (ORFs), repressing its own expression as well as that of Rep, viral replication, and leaf development and senescence (Gutierrez et al., 2004; Hefferon et al., 2006). Recently, RepA was reported to be encoded by oat dwarf virus (ODV), bean yellow dwarf virus (BYDV), and mulberry mosaic dwarf-associated virus (MMDaV) and found to have the ability to induce HR-like cell death in *Nicotiana benthamiana* (Qian et al., 2016; Diamos & Mason, 2019; Sun et al., 2022).

Citrus chlorotic dwarf-associated virus (CCDaV) is a member of the genus *Citlodavirus* and was first observed in Turkey in the 1980s (Kersting et al., 1996). The host range of CCDaV is restricted to citrus and related species (Yang et al., 2022) and has been found in China and Thailand, where it has caused significant losses to lemon and pummelo yields (Guo et al., 2015; Yang et al., 2020; Yang et al., 2022). The genome of CCDaV ranges from 3639 to 3763 nucleotides, including four ORFs that encode the predicted coat proteins V1, V2, and V3, and the putative movement protein V4 in the virion strand, as well as RepA (C1) and Rep (C2) on the complementary strand (Roumagnac et al., 2022).

Because CCDaV is a newly discovered virus and studying woody plant-virus interactions is complex, the function of CCDaV-RepA and its effect on molecular and physiological responses in the host plant have not been characterized. To identify the interaction between CCDaV-RepA and plant defense responses. In this study, RepA was transiently expressed in *N. benthamiana*, and induced a HR-like cell death. The ability of RepA to localize to the nucleus was required for inducing a HR-like cell death. Further study suggested that *WRKY1* could positively regulate RepA-induced cell death.

## 2 Results

### 2.1 CCDaV-encoded RepA and Rep induce HR-like cell death in *N. benthamiana*

**Figure 1:**
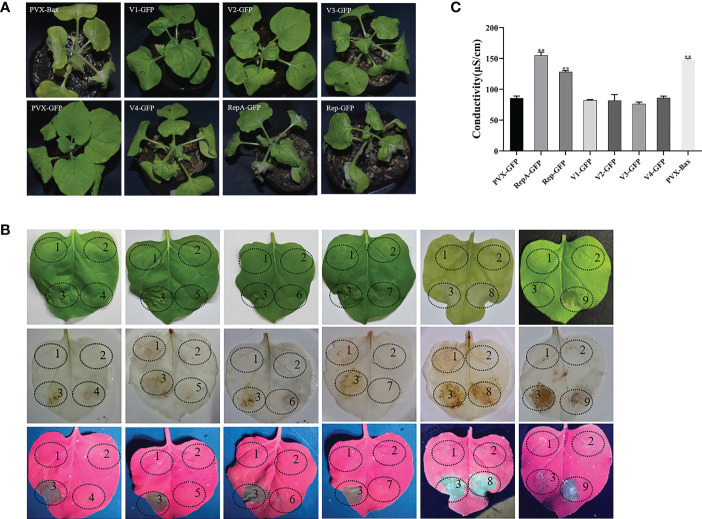
Identification of cell death induced by CCDaV-encoded proteins in *Nicotiana benthamiana*. **(A)** Disease symptoms observed in *N. benthamiana* leaves infiltrated with PVX-Bax, PVX-GFP, or recombinant PVX-GFP vectors expressing individual CCDaV proteins at 5 days post-infiltration (dpi). **(B)** CCDaV-encoded proteins transiently expressed in *N. benthamiana*. Photos were taken under white light (top panels); after 3,3′-diaminobenzidine staining (middle panels); and under ultraviolet light at 5 dpi (bottom panels). 1, PVX-GFP; 2, Buffer; 3, PVX-Bax; 4, V1-GFP; 5, V2-GFP; 6, V3-GFP; 7, V4-GFP; 8, RepA-GFP; 9, Rep-GFP. **(C)** Conductivity detection at 3 dpi. Significant differences (**p < 0.01) were determined by Student’s t test. Each experiment was repeated four times with at least six independent biological replicates.

To evaluate whether CCDaV-encoded proteins induce HR-like cell death, all six proteins encoded by CCDaV were transiently expressed in *N. benthamiana* using the potato virus X (PVX) vector carrying the green fluorescent protein (PVX-GFP). At 4 days post-infiltration (dpi), RepA-GFP and Rep-GFP induced cell death in infiltrated leaves, which was similar to the HR-based cell death triggered by the pro-apoptotic mouse BCL2-associated X protein (PVX-Bax) positive control (**Figure 1A**). Severe necrosis and collapse symptoms were observed in *N. benthamiana* emerging leaves that were infiltrated with RepA-GFP and Rep-GFP at 7 dpi and 9 dpi, respectively. No necrosis was found in *N. benthamiana* infiltrated with the strain GV3101 pSOUP carrying V1-GFP, V2-GFP, V3-GFP, V4-GFP, or PVX-GFP after 13 dpi (**Figure 1A**). To exclude the possible effect of PVX on cell death activity induced by CCDaV-encoded proteins, all six CCDaV proteins were also transiently expressed using the pNmGFPer vector (kindly provided by Prof. Xiuping Zou, Southwest University, China) in *N. benthamiana.* At 9 dpi, CCDaV-Rep and RepA-induced HR-like cell death in *N. benthamiana* leaves (**Figure S2**), while the other four CCDaV proteins did not trigger these symptoms up to 13 dpi.

To exclude the possible influence of GFP on cell death-inducing activity, the six CCDaV proteins were transiently expressed using the PVX vector into *N. benthamiana*. The results showed that only Rep and RepA could induce cell death in the infiltrated leaves, with no significant difference found in the titer of PVX or PVX derivatives infiltrated into *N. benthamiana* (**Figure S1**).

The accumulation of ROS (i.e., H_2_O_2_) and increased ion leakage are typical features of HR (Mur et al., 2008). At 5 dpi, RepA-GFP- and Rep-GFP-infiltrated *N. benthamiana* leaves typically stained brown after 3,3′-diaminobenzidine (DAB) treatment, indicating that RepA and Rep induced peroxidase activity and subsequent H_2_O_2_ production (**Figure 1B**). To quantify cell death, electrolyte leakage was also analyzed. The results showed that the conductivity of *N. benthamiana* leaves infiltrated with RepA-GFP or Rep-GFP was significantly higher (*p* < 0.001) than that of *N. benthamiana* infiltrated with other GFP-fusion structures (V1-GFP, V2-GFP, V3-GFP, V4-GFP, and PVX-GFP) at 3 dpi (**Figure 1C**). Taken together, CCDaV-RepA and Rep elicited HR-like cell death in *N. benthamiana*. Considering that RepA produced more severe symptoms than Rep, RepA was selected for subsequent analyses.

### 2.2 Mapping the key domains for RepA-induced HR-like cell death

**Figure 2:**
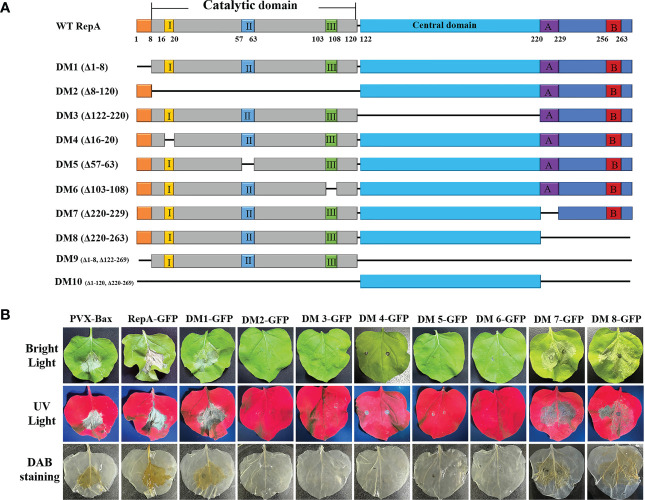
Mapping the key domains in CCDaV-RepA associated with HR-like cell death. **(A)** Schematic representation of RepA and its derivatives used in this study. I, II, and III indicate the conserved motifs in the catalytic domain contains. A and B indicate the Walker A and B motifs, respectively. **(B)** RepA-GFP and RepA^DM1-8^-GFP were transiently expressed in *N. benthamiana* and imaged under bright light (top panels), ultraviolet light (middle panels), and after staining with 3,3′-diaminobenzidine (DAB) (bottom panels) at 5 days post-infiltration (dpi). All sample measurements were repeated four times.

The functional domains of CCDaV-RepA were predicted using SMART (http://smart.embl-heidelberg.de/smart/set_mode.cgi?NORMAL=1) and Interpro (http://www.ebi.ac.uk/inter) software (Sun et al., 2022), and seven conserved domains were predicted in RepA (**Figure 2A**). To explore the key domain(s) associated with RepA-induced HR-like cell death, eight truncated proteins were transiently expressed in *N. benthamiana*. The results demonstrated that RepA^DM1^-GFP (amino acid 1-8 deleted), RepA^DM7^-GFP (amino acid 220-264 deleted), and RepA^DM8^-GFP (amino acid 1-8, 122-263 deleted) could still produce HR-like cell death and H_2_O_2_ in *N. benthamiana* similar to that of the full-length RepA-GFP. However, the three rolling-circle replication motif-deletion mutants, RepA^DM4^-GFP (amino acid 16-20 deleted), RepA^DM5^-GFP (amino acid 57-63 deleted), and RepA^DM6^-GFP (amino acid 103-108 deleted), as well as RepA^DM2^-GFP without the catalytic domain (amino acid 8-120 deleted) or RepA^DM3^-GFP without the central domain (amino acid 122-220 deleted), produced leaf curling in the infiltrated *N. benthamiana* and no H_2_O_2_ deposition was observed (**Figure 2B**). These results confirmed that the rolling-circle replication motifs of CCDaV-RepA were associated HR-like cell death induction in *N. benthamiana*.

### 2.3 Subcellular localization of RepA and its deletion mutants

**Figure 3:**
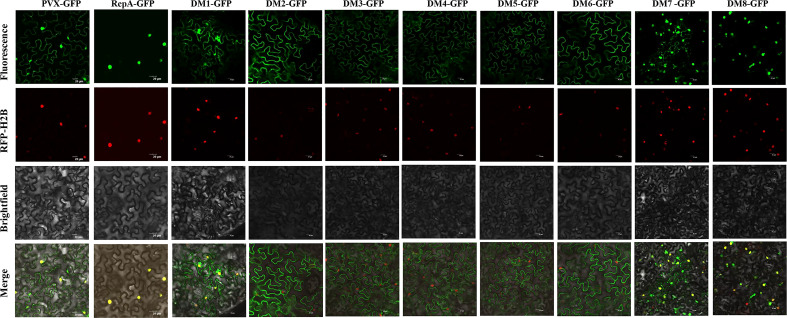
Subcellular localization analyses of CCDaV-RepA and its derivatives in *Nicotiana benthamiana* epidermal cells. Red fluorescent protein-histone 2B (RFP-H2B) was co-infiltrated with PVX-GFP or PVX-GFP-fusion constructs into *N. benthamiana* leaves. Each experiment was repeated four times and at least 40 cells (average 10 cells per field) were observed per plant. Scale bars = 20 µm.

Identification of the subcellular localization of a viral protein is an important step in understanding its putative functions (Zhan et al., 2018; Diamos & Mason, 2019). Confocal microscopy analysis suggested that RepA-GFP localized exclusively in the nucleus of epidermal cells at 48 h post-inoculation (hpi). As with RepA-GFP, RepA^DM1^-GFP, RepA^DM7^-GFP, and RepA^DM8^-GFP localized in the nucleus at 28-36 hpi. However, RepA^DM2^-GFP, RepA^DM3^-GFP RepA^DM4^-GFP, RepA^DM5^-GFP, and RepA^DM6^-GFP were predominantly found in the cytoplasm (**Figure 3**). These results showed that the first eight amino acids at the N terminus, amino acids 122-263, and amino acids 220-264 of RepA were not associated with its nuclear localization. Furthermore, no basic amino acids with a consensus nuclear localization signal sequence were predicted using the PSORT online software, indicating the presence of an atypical nuclear localization signal.

### 2.4 RepA elicits plant immune responses in *N. benthamiana*

**Figure 4:**
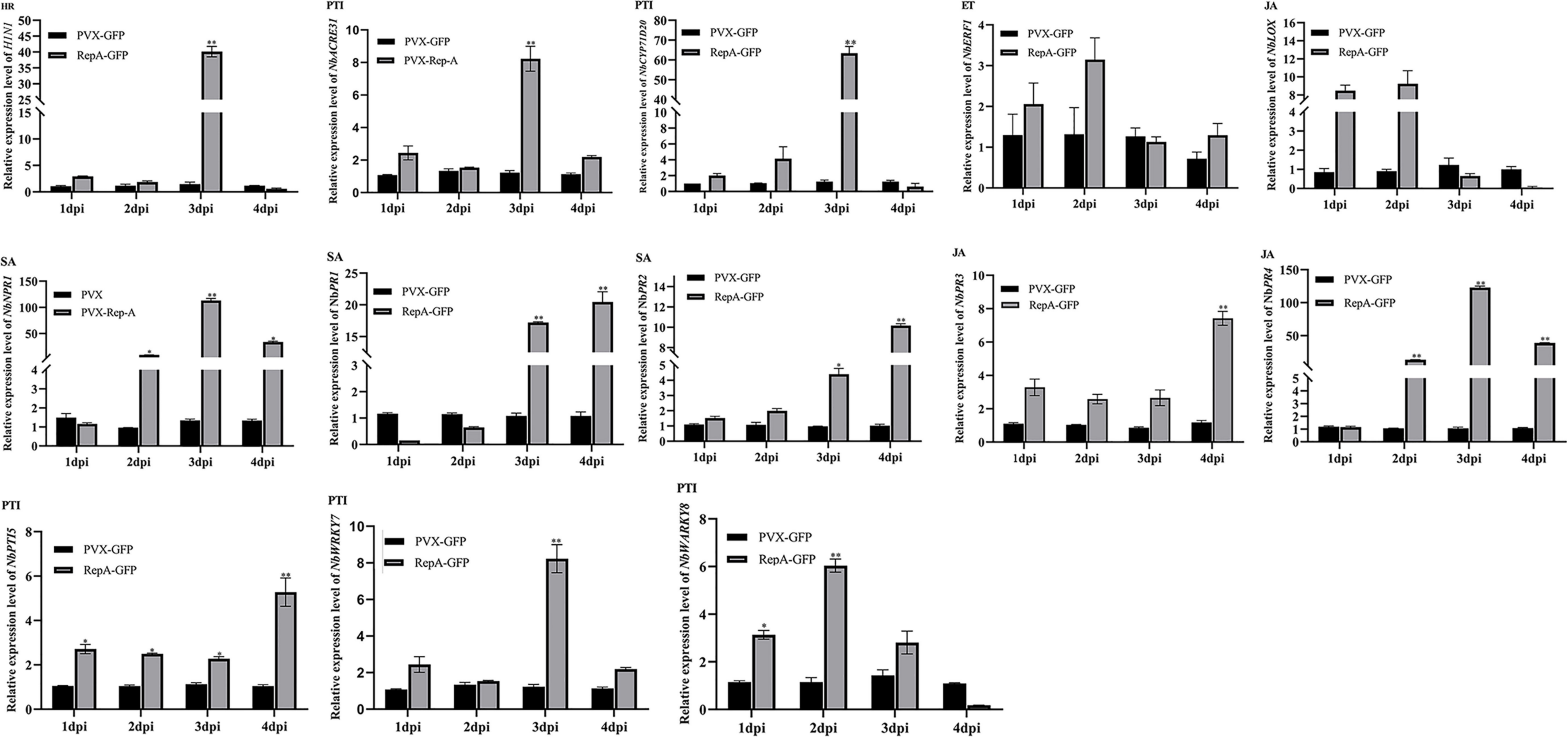
Changes in the expression level of defense-related genes in *Nicotiana benthamiana* leaves inoculated with PVX-GFP or RepA-GFP. *NbACTIN* was used as the housekeeping gene. The expression of each gene was quantified using the 2^−ΔΔCt^ method. Statistical differences were calculated with the Student’s t-test, where *p < 0.05 and **p < 0.01. The experiment was repeated using four independent biological replicates and four technical replicates.

The innate immune system of plants has an interconnected two-layered system of pathogen-associated molecular patterns (PAMP)-triggered immunity (PTI) and effector-triggered immunity (ETI), with the JA and SA signaling pathways also playing roles in plant defense (Jones & Dangl, 2006; Zhang et al., 2022). To further determine the association between RepA-induced cell death and plant immune responses, reverse transcription-quantitative polymerase chain reaction (RT-qPCR) was used to quantify the key defense response-related genes involved in the SA (*NbPR1*, *NbPR2*), JA (*NbPR3*, *NbPR4* and *NbLOX*), and ET (*NbERF1*) pathways, as well as marker genes for HR (*HIN1*) and PAMP-triggered immunity (*NbWRKY7*, *NbWRKY8*, and *NbACRE31*) (Du et al., 2022; Sun et al., 2022). The results showed that the levels of *NbPR1*, *NbPR2*, *NbPR3*, *NbPR4*, *NbWRKY7*, *NbWRKY8*, and *NbACRE31* were significantly upregulated in *N. benthamiana* infiltrated with RepA-GFP, with a 4-100-fold increase at 3 dpi when compared with the negative control. Furthermore, the expression of *NbNPR1*, which is the upstream regulator of the SA signaling pathway, also increased (**Figure 4**). These results showed that CCDaV-RepA induced HR-related plant defense responses.

### 2.5 Other CCDaV-encoded proteins do not prevent CCDaV-RepA-induced HR-like cell death

**Figure 5:**
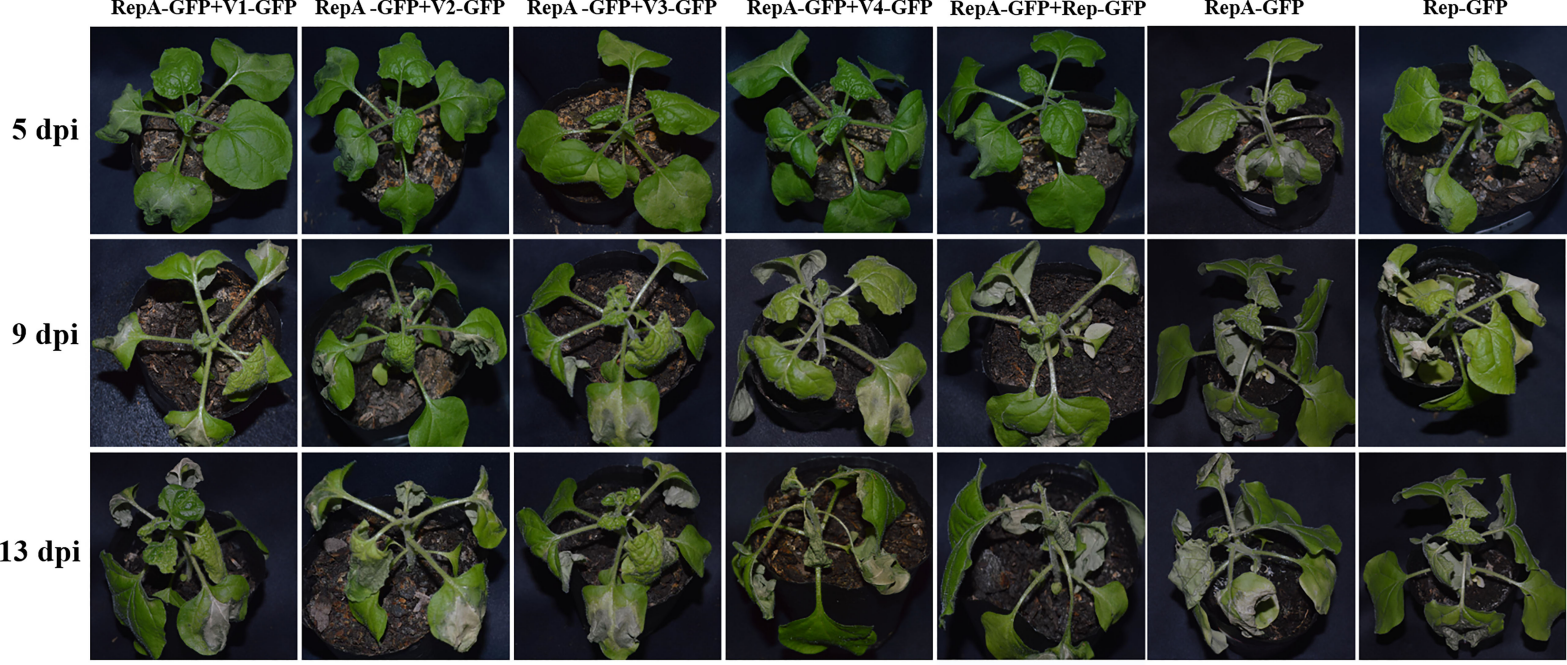
CCDaV-encoded proteins inhibited cell death induced by RepA. Each experiment was repeated three times and at least five plants were used per biological replicate.

Several studies showed that some plant virus-encoded proteins can inhibit HR-induced cell death to support virus survival (Mubin et al., 2010). However, the cell death symptoms in *N. benthamiana* produced by the co-expression of RepA with other CCDaV-encoded proteins (V1, V2, V3, V4, and Rep) were the same as those produced by RepA alone (**Figure 5**). Furthermore, no significant difference in the accumulation of RepA among the different inoculated leaves was observed.

### 2.6 *WRKY1* positively regulates RepA-induced cell death in *N. benthamiana*

**Figure 6:**
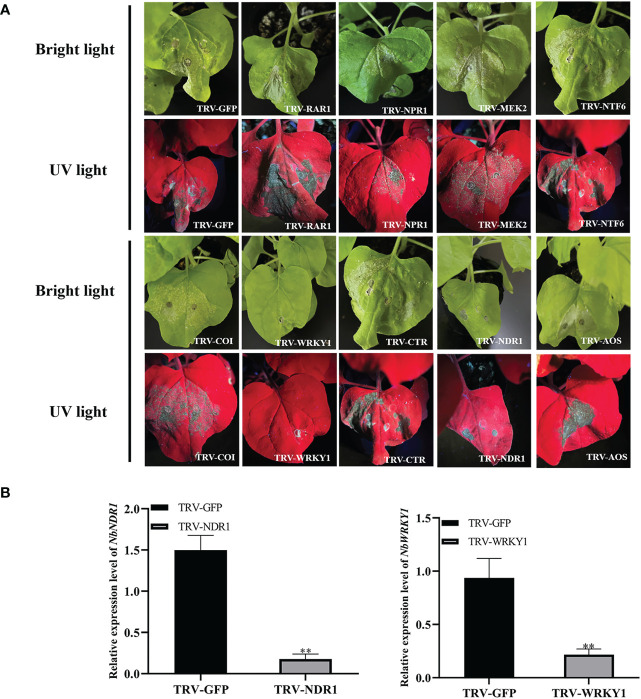
Effect of signaling cascade silencing on RepA-induced cell death in *Nicotiana benthamiana*. **(A)** Disease symptoms in silenced *N. benthamiana* plants at 3 days post-infiltration (dpi) with RepA-GFP. **(B)** The expression level of *NbWRKY1* was calculated using the 2^−ΔΔCt^ method with the housekeeping gene *NbACTIN* (Student’s t-test, **p < 0.01). Each experiment was repeated four times and at least five plants were used per biological replicate.

Previous studies showed that signaling cascade components, such as *WRKY1*, *NPR1*, *AOX, COI*, *CTR*, *NDR1*, *RAR1*, *NTF6*, and *MEK2* play important roles in cell death (Zhang et al., 2012; Meng & Zhang, 2013; Adachi et al., 2016). Therefore, these signaling cascade components were silenced by using the tobacco rattle virus (TRV) vector in *N. benthamiana* to assess whether they are involved in RepA-induced cell death. At 9 dpi, RepA-GFP was infiltrated in the emerging leaves of the silenced plants (**Figure 6A**). RT-qPCR analysis showed that the relative transcript level of *RAR1*, *COI*, *CTR*, *NTF6*, *NPR1*, *MEK2*, and *AOX* in silenced *N. benthamiana* plants was reduced by approximately 62.68 - 92.62% (**Figure S4**) in these plants, which exhibited significant cell death similar to that induced by RepA alone. The relative transcript level of *WRKY1* and *NDR1* in silenced *N. benthamiana* plants was reduced by approximately 88.35% and 88.14%, respectively (**Figures 6B, C**). Conversely, no apparent cell death was observed in *WRKY1-*silenced plants after RepA was transiently expressed, while *NDR1-*silenced *N. benthamiana* produced mild cell death symptoms.

### 2.7 *NbWRKY1* is upregulated by RepA

**Figure 7:**
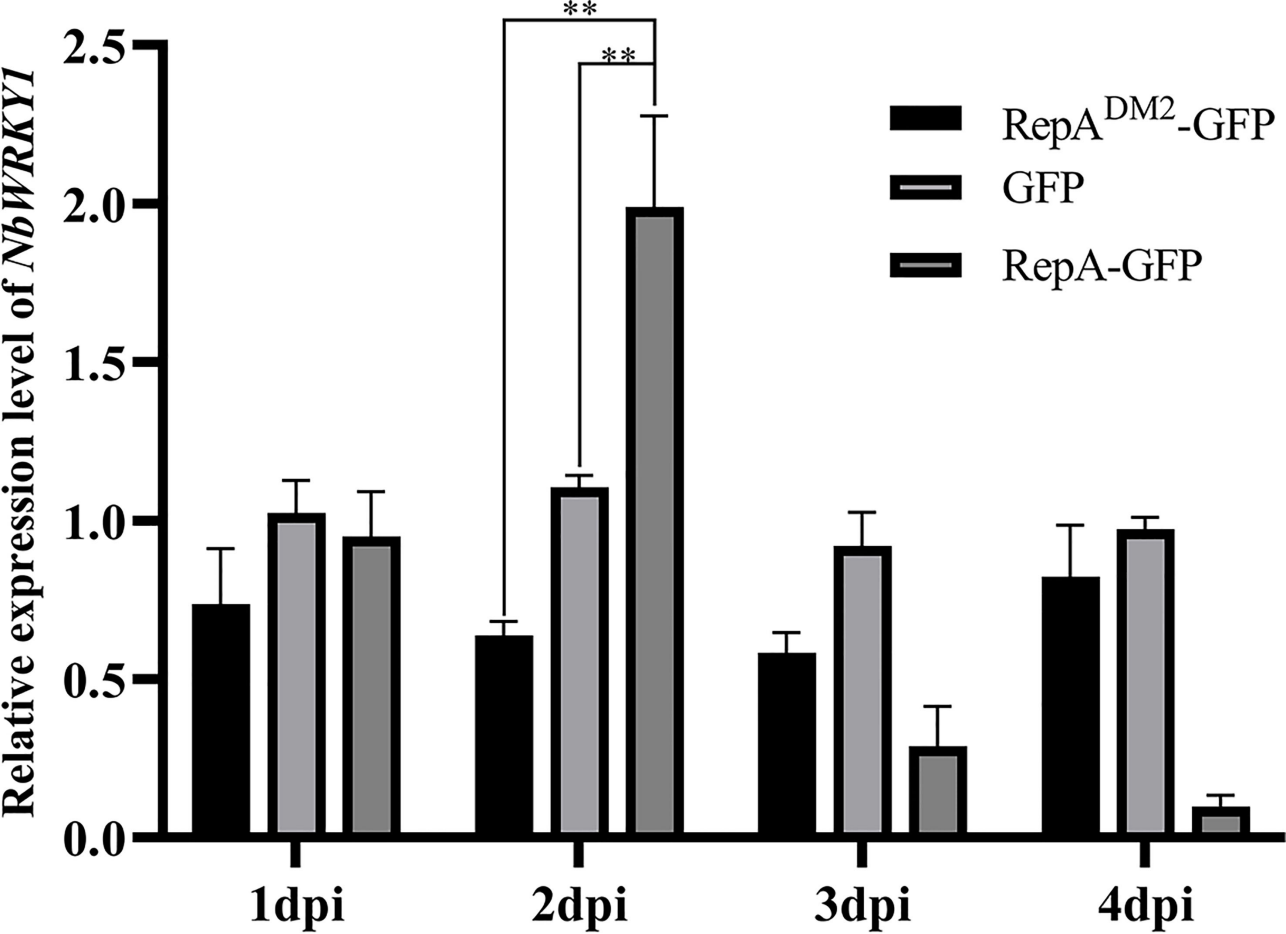
Relative expression of *NbWRKY1* in response to transient expression of RepA^DM2^-GFP, GFP, and RepA-GFP. Total RNA extracted at 4 days post-infiltration (dpi) was used for reverse transcription quantitative PCR (RT-qPCR) analysis. Significant differences (**p < 0.01) were determined with the Student’s t-test. The experiment was repeated four times with four independent biological replicates.

At 2 dpi, *NbWRKY1* transcripts were increased approximately 2.78-fold and 2.17-fold in *N. benthamiana* infiltrated with PVX-RepA compared to the control plants infiltrated with RepA^DM2^-GFP and PVX-GFP, respectively (**Figure 7**). These results suggested that CCDaV-RepA upregulated the expression of *NbWRKY1*.

As NbWRKY1 and RepA localize to the nucleus (Sun et al., 2022), yeast two-hybrid (Y2H), firefly luciferase complementation imaging (LCI), and bimolecular fluorescence complementation (BiFC) assays were used to determine whether there was a direct interaction between NbWRKY1 and CCDaV-RepA. However, the results showed RepA could not directly interact with WRKY1.

## 3 Discussion

RepA is a multifunctional protein with an essential role in the viral DNA replication of geminiviruses (Varsani et al., 2017). In addition, RepA is a pathogenicity factor and potent inducer of HR in plants in some geminiviruses, including soybean geminivirus A, MMDaV, and ODV (Dodds & Rathjen, 2010; Qian et al., 2016; Yang et al., 2018; Sun et al., 2022). In this study, transient expression of CCDaV-RepA induced severe necrosis and collapse in *N. benthamiana*, indicating its function as a potential key virulence factor. Furthermore, CCDaV-RepA elicited HR-like cell death in *N. benthamiana*. HR is generally associated with the recognition of specific pathogen-encoded Avr proteins produced by the plant-encoded *R* gene product (Martin et al., 2003). Therefore, CCDaV-RepA may act as an *Avr* gene and be recognized by an unknown host *R* gene product that elicits a defense reaction that can potentially attenuate virus infection.

Previous reports revealed that deletion of the N-terminal amino acid compromises the ability of ODV-RepA to induce HR-like cell death in *N. benthamiana* (Qian et al., 2016). In contrast, in this study, only the rolling-circle replication motifs were associated with HR-like cell death, which was more consistent with findings reported for MMDaV (Sun et al., 2022). These results reconfirmed that not all positional homologs of geminivirus-encoded proteins are made equal (Luna & Lozano-Durán, 2020).

Previous studies showed that the nuclear localization of the transcription activation protein of tomato yellow leaf curl China virus (TYLCV), East African cassava mosaic virus (EACMV), and the RepA of MMDaV was essential for inducing HR-like cell death (Dong et al., 2003; Chowda-Reddy et al., 2009; Sun et al., 2022). In this study, nuclear localization of CCDaV-RepA was involved in its induction of HR-like cell death. Furthermore, sequence prediction results suggested that the CCDaV-RepA protein may contain an atypical nuclear localization signal. Considering RepA may be transported to the nucleus through protein-protein interaction, further research will be carried out in the future.

The WRKY family is one of the largest transcription factor (TF) families in plants (Yue et al., 2016). Although several studies have elucidated the role of WRKY TFs in bacterial and fungal pathogen responses (Hu et al., 2012; Kim et al., 2014; Abbas et al., 2020), very few plant virus-responsive WRKYs have been reported. Some studies showed that WRKY TFs positively regulated the defense response to tobacco mosaic virus (TMV) and *cucumber mosaic virus* (CMV) infection, and participated in disease resistance through the transcriptional reprogramming of pathogenesis-related gene expression (Huh et al., 2015; Zou et al., 2019). While WRKY8 and MeWRKY81 are involved in the negative regulation of crucifer-infecting TMV (TMV-cg) and South African cassava mosaic virus (SACMV), respectively (Chen et al., 2013; Freeborough et al., 2021). In this study, the expression of *NbWRKY1* was upregulated by the expression of CCDaV-RepA in *N. benthamiana*. Moreover, silencing *WRKY1* inhibited RepA-induced cell death, indicating that *Nb*WRKY1 is important for RepA-induced cell death by regulating the downstream genes associated with antiviral defense. NbWRKY1 was reported to be located in nuclear (Sun et al., 2022). However, no direct interaction between NbWRKY1 and CCDaV-RepA was found using multiple interaction assays. These results indicated that an indirect interaction may exist between CCDaV-RepA and NbWRKY1.

In conclusion, CCDaV-RepA elicited HR-like cell death and plant immune responses in *N. benthamiana*. RepA upregulated the expression level of *NbWRKY1* and *NbWRKY1*, which positively regulated CCDaV-RepA-induced cell death. These findings provide novel insight into the interactions between CCDaV and the host plant. In the future, the role of the NbWRKY1 regulatory network in the antiviral response of citrus will be elucidated.

## 4 Materials and methods

### 4.1 Virus source and plant material

The CCDaV isolate ULK-1 was maintained on Eureka lemon (*C. limon*) and used as the virus source for all experiments. *N. benthamiana* plants were grown in an incubator at 25°C under a 16:8 h light/dark cycle.

### 4.2 Expression constructs

The PVX-GFP-containing pGR106 vector (N-terminal GFP-fusion) was constructed as described previously (Earley et al., 2006). Full-length *RepA, Rep, V1*, *V2, V3*, and *V4* genes encoded by CCDaV were amplified by using specific primer pairs (**Table S1**) and individually cloned into the pGEM-T Easy vector (Promega). These plasmids were digested using *Cla* I and *Sal* I, and ligated into PVX-GFP to generate RepA-GFP, Rep-GFP, V1-GFP, V2-GFP, V3-GFP, and V4-GFP. The PVX-V1, PVX-V2, PVX-V3, PVX-V4, PVX-RepA, and PVX-Rep clones were constructed as described by Yang et al. (2018).

To explore the key domain(s) of RepA, eight deletion mutations were amplified (**Table S1**) and cloned into PVX-GFP to yield RepA^DM1^-GFP to RepA^DM8^-GFP by homologous recombination (Sun et al., 2022). All constructs were verified by sequencing.

### 4.3 *Agrobacterium*-mediated infiltration

All constructs were transformed into *Agrobacterium tumefaciens* GV3101 as described by Du et al. (2022). The *A. tumefaciens* cultures harboring the constructs were adjusted to a final OD_600_ 0.6-0.8 and incubated in the dark for 2-3 h before infiltration. The suspensions were then infiltrated into at least six fully expanded leaves of four-week-old *N. benthamiana*.

### 4.4 H_2_O_2_ detection in *N. benthamiana*

*N. benthamiana* leaves were collected at 5 dpi and soaked in 1 mg/mL DAB-HCl solution (pH 3.8). The samples were incubated in the dark for 8 h at room temperature and then bleached in boiling 96% ethanol for 10 min (Hou et al., 2014).

### 4.5 Measurement of electrolyte leakage

Five *N. benthamiana* leaf discs (9 mm in diameter) were collected from infiltrated areas at 3 dpi. The tissues were incubated in 10 mL distilled water at 28°C for 1 h (Pitino et al., 2016). The solution conductivity was measured using an FE30 conductivity meter (Mettler-Toledo Company), and the electrolyte leakage was calculated by the ratio of the conductivity to total conductivity.

### 4.6 Protein extraction and western blot analysis

The total protein from infiltrated *N. benthamiana* leaves exhibiting transient expression was extracted using a Plant Total Protein Extraction Kit (Solarbio). Western blot analysis was performed as described previously (Du et al., 2022) with an anti-GFP monoclonal primary antibody (Proteintech) and anti-mouse IgG coupled with horseradish peroxidase (HRP) (Proteintech) as primary and secondary antibodies, respectively. The products were visualized using a chemiluminescence detection reagent (Everbright).

### 4.7 Enzyme-linked immunosorbent assay

Systemic leaf samples of *N. benthamiana* infiltrated with PVX or derivatives of PVX were detected using ELISA as previously described (Wang et al., 2014; Qian et al., 2016).

### 4.8 Laser scanning confocal microscopy

For subcellular localization, mCherry-H2B and RFP-plasma membrane were used as nuclear and plasma membrane markers, respectively. At 48 hpi, fluorescence signals of the epidermal cells from infiltrated *N. benthamiana* leaves were observed using an FV3000 confocal microscope (Olympus) (Du et al., 2022).

### 4.9 Virus-induced gene silencing assay in *N. benthamiana*

The TRV silencing vector was kindly provided by Dr. Wanxia Shen from the Southwest University, China and used for the VIGS assay in *N. benthamiana* previously described (Senthil-Kumar & Mysore, 2014). *NbWRKY1*, *NbNDR1*, *NbNPR1*, *NbCOI1*, *NbCTR1*, *NbNTF6*, *NbRAR1*, and *NbMEK2* were amplified (**Table S1**) and cloned into TRV2. After sequencing verification, TRV1 and its derivatives were co-inoculated into *N. benthamiana* using GV3101 infiltration (Sun et al., 2022).

### 4.10 Total RNA extraction and RT-qPCR analysis

To evaluate the silencing efficiency, total RNA was extracted from systemically infected leaves of *N. benthamiana* at 10 dpi using TRIzol (Tiangen). To quantify the expression of host defense-related genes responsive to CCDaV-RepA, total RNA was extracted from infiltrated leaves at 3 dpi. RT-qPCR was performed using The BlasTap™2×RT-qPCR Master MIX (ABM). The housekeeping gene *NbActin* was used as an internal control for RNA quantification (Ananthakrishnan et al., 2010). Each experiment included samples run in triplicate, including the internal control gene. The expression of target genes was calculated using the formula described by Pfaffl (2001). The primers for RT-qPCR are listed in **Table S1**.

### 4.11 Yeast-two-hybrid assay

The Matchmaker Yeast Two-Hybrid System (Weidi Biotechnology) was used for Y2H assays to verify the interaction between RepA and NbWRKY1. The Y2H screening was performed as described previously (Shi et al., 2007).

### 4.12 Firefly luciferase complementation imaging assay

The full-length RepA and NbWRKY1 were amplified with specific primers (**Table S1**) and inserted into PCCL-P9 and PHNL vectors, which were kindly provided by Professor Jianmin Zhou, University of Chinese Academy of Sciences, Beijing, to produce PCCL-P9-CCDaV-RepA and PHNL-NbWRKY1, respectively. The constructs were introduced into *A. tumefaciens* GV3101 pSOUP and agroinfiltrated into *N. benthamiana* leaves. The infiltrated leaves were detached at 48 hpi and sprayed with 1 mM luciferin (Macklin). The tissues were kept in dark for 6 min before the LUC activity was detected by using IVIS Lumina Series III system (PerkinElmer).

### 4.13 Bimolecular fluorescence complementation assay

RepA and NbWRKY1 were amplified with specific primers (**Table S1**) and cloned into pSPYCE-35S and pSPYNE-35S, respectively. After sequencing verification, the recombinant vectors were transformed into *A. tumefaciens* GV3101 pSOUP and infiltrated to *N. benthamiana* leaves at the four- to six-leaf stages. At 48 hpi, the fluorescence signal was observed by FV3000 scanning confocal microscope (Olympus).

### 4.14 Statistical analyses

GraphPad Prism 9 was used for statistical analyses, and the data were expressed as the means ± standard deviation (SD) of three biological replicates. Significant differences in gene expression were evaluated using Student’s test at *P < 0.05, **P < 0.01.

## Data availability statement

The original contributions presented in the study are included in the article/**Supplementary Material**. Further inquiries can be directed to the corresponding author.

## Author contributions

YQ, CZ, and YZ designed the experiments. YQ, XY, and JZ performed the experiments. YQ and JW analyzed the data, and YQ wrote the draft manuscript. YZ revised and polished the manuscript. All authors contributed to the article and approved the submitted version.

## Supplementary material

Supplementary Figure 1 Detection of *Potato virus X* (PVX) content after RepA from the citrus chlorotic dwarf-associated virus was transiently expressed in *Nicotiana benthamiana* using the PVX vector. The error bars indicate the standard deviation within one representative experiment (n = 3). NG indicates uninoculated *N. benthamiana* plants. Each experiment was repeated three times and at least five plants were used per biological replicate.

Supplementary Figure 2 Identification of cell death induced by CCDaV-encoded proteins in *Nicotiana benthamiana* by using pNmGFPer vector. **(A)** Disease symptoms observed in *N. benthamiana* leaves expressing individual CCDaV proteins at 9 days post-infiltration (dpi). **(B)** Images of infiltrated leaves were captured under bright light and UV light at 9 dpi. The 3,3′-diaminobenzidine (DAB) staining was performed to visualize the production of H_2_O_2_. Each experiment was repeated three times and at least five plants were used per biological replicate.

Supplementary Figure 3 Mapping the key domains of CCDaV-RepA associated with HR-like cell death. 35S-RepA-GFP, 35S-RepA^DM1^-GFP, 35S-RepA^DM2^-GFP, 35S-RepA^DM7^ -GFP, and 35S-RepA^DM8^-GFP were transiently expressed in *N. benthamiana* and then imaged under bright light (top panels), ultraviolet light (middle panels), and after 3,3′-diaminobenzidine (DAB) treatment (bottom panels) at 9 days post-infiltration.

Supplementary Figure 4 Effect of signaling cascade component silencing on RepA-induced cell death in *Nicotiana benthamiana*. The expression of *NbWRKY1* was calculated using the formula 2 ^−ΔΔCt^ with the housekeeping gene, *NbACTIN* (Student’s t-test ***p* < 0.01).
