# Supplementary figures and images for: Regulation of Nicotiana benthamiana cell death induced by citrus chlorotic dwarf-associated virus-RepA protein by WRKY 1
Source: Front Plant Sci. 2023 Apr 25;14:1164416. doi: 10.3389/fpls.2023.1164416 (PMC10167294; doi:10.3389/fpls.2023.1164416)

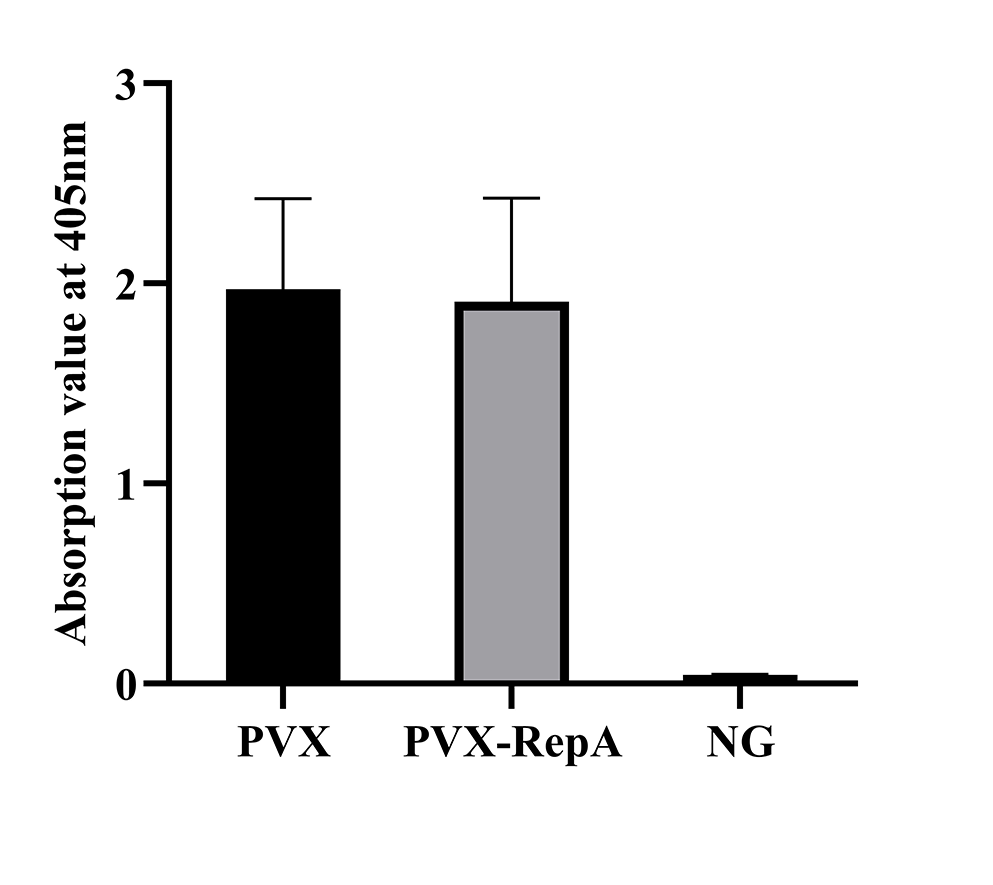

Supplement: Supplementary Figure 1 — Detection of Potato virus X (PVX) content after RepA from the citrus chlorotic dwarf-associated virus was transiently expressed in Nicotiana benthamiana using the PVX vector. The error bars indicate the standard deviation within one representative experiment (n = 3). NG indicates uninoculated N. benthamiana plants. Each experiment was repeated three times and at least five plants were used per biological replicate. [file Image_1.tif]

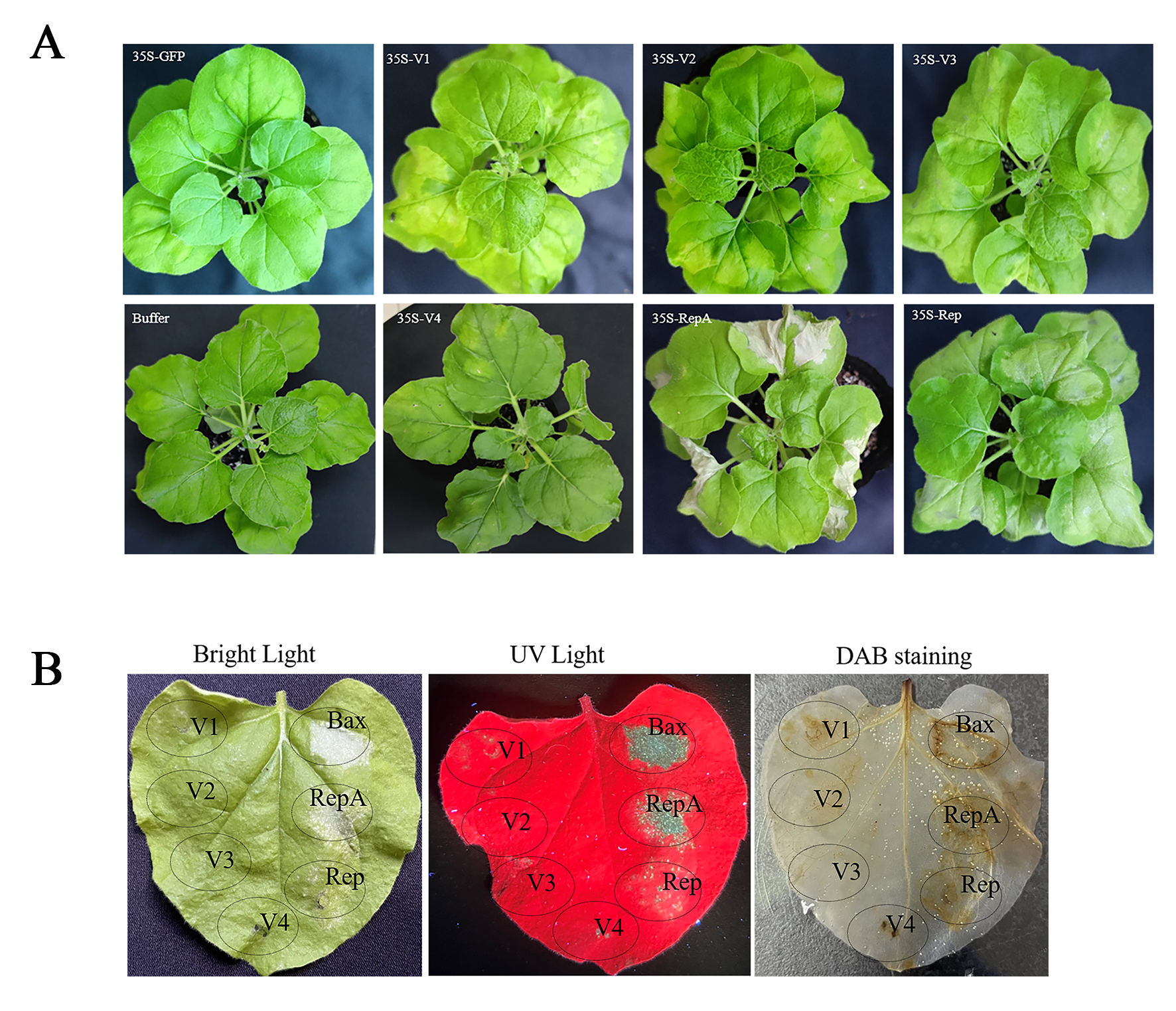

Supplement: Supplementary Figure 2 — Identification of cell death induced by CCDaV-encoded proteins in Nicotiana benthamiana by using pNmGFPer vector. (A) Disease symptoms observed in N. benthamiana leaves expressing individual CCDaV proteins at 9 days post-infiltration (dpi). (B) Images of infiltrated leaves were captured under bright light and UV light at 9 dpi. The 3,3′-diaminobenzidine (DAB) staining was performed to visualize the production of H2O2. Each experiment was repeated three times and at least five plants were used per biological replicate. [file Image_2.tif]

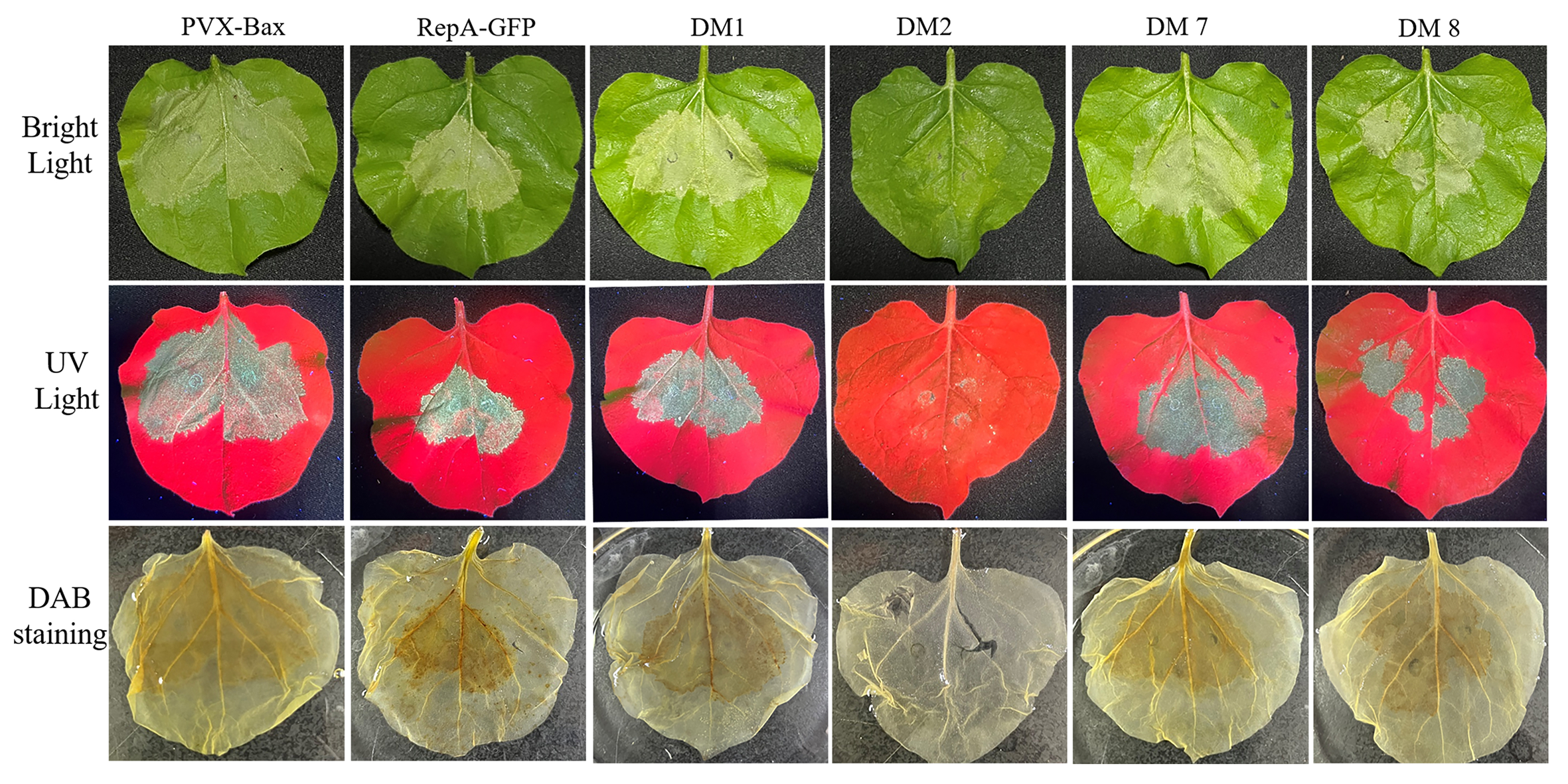

Supplement: Supplementary Figure 3 — Mapping the key domains of CCDaV-RepA associated with HR-like cell death. 35S-RepA-GFP, 35S-RepADM1-GFP, 35S-RepADM2-GFP, 35S-RepADM7 -GFP, and 35S-RepADM8-GFP were transiently expressed in N. benthamiana and then imaged under bright light (top panels), ultraviolet light (middle panels), and after 3,3′-diaminobenzidine (DAB) treatment (bottom panels) at 9 days post-infiltration. [file Image_3.tif]

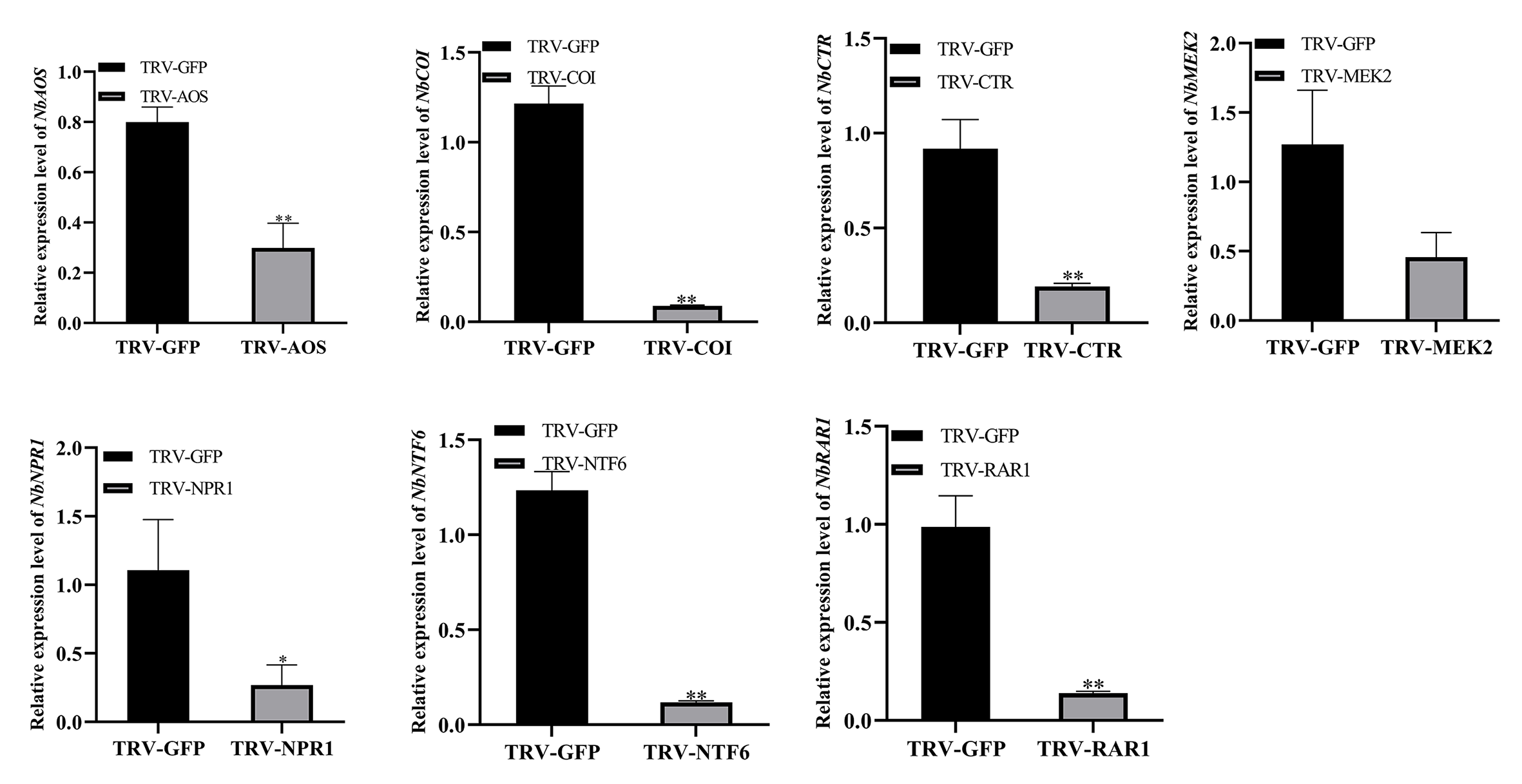

Supplement: Supplementary Figure 4 — Effect of signaling cascade component silencing on RepA-induced cell death in Nicotiana benthamiana. The expression of NbWRKY1 was calculated using the formula 2 −ΔΔCt with the housekeeping gene, NbACTIN (Student’s t-test **p < 0.01). [file Image_4.tif]
