# Supplementary material for: Regulation of Nicotiana benthamiana cell death induced by citrus chlorotic dwarf-associated virus-RepA protein by WRKY 1
Source: Front Plant Sci. 2023 Apr 25;14:1164416. doi: 10.3389/fpls.2023.1164416 (PMC10167294; doi:10.3389/fpls.2023.1164416)
Supplement: Supplementary file 5 [file Table_1.docx]

**Table S1 Primers used in this study**

| Primers | Sequences (5'-3') |
| --- | --- |
| **For cloning of CCDaV proteins by pGR106 vectors** | |
| V1-F | CACCAGCTAGCATCGATATGGTGAGTACCAGGAGTGGAG |
| V1-R | GCTTATCGGCGGTCGACttaATTTGATGTAGAATCATAAAAATA |
| V2-F | CACCAGCTAGCATCGATATGTGTCATTATGCATTAAGTGTTCAAGATTTGC |
| V2-R | GCTTATCGGCGGTCGACttaCACCCCGGAGGAACACC |
| V3-F | CACCAGCTAGCATCGATATGAAACGTGTTGGGCACGT |
| V3-R | CTTATCGGCGGTCGACttaCCCAGGGCTCCTCCTACGGG |
| V4-F | CACCAGCTAGCATCGATATGGACGGTCAAGATTTGGTGTTACA |
| V4-R | GCTTATCGGCGGTCGACttaAGCCAAGCTTTTTTTCTTA |
| C1/C2-F | CACCAGCTAGCATCGATATGGCTTCCACTTCCTCTAGCTT |
| C1-R | AGCTTATCGGCGGTCGACctaAACTTCTGGCCCAGGCG |
| C2-R | TTATCGGCGGTCGACttaGTAGAATACATCAGT |
| **For the construction of RepA mutants** | |
| PVX-DM1-F | ATGGACGAGCTGTACAAGcccgggatgCGATTCTCAGCCAAAAATAT |
| PVX-DM1-R | CTTATCGGCGGTCGACCTAAACTTCTGGCCCAGGCG |
| PVX-DM2-F | GCATGGACGAGCTGTACAAGcccgggATGGCTTCCACTTCCTCTAGCTTCCGATCCCCACAAAAG |
| PVX-DM2-R | CTATCAAGCTTATCGGCGGTCGACCTAAACTTCTGGCCCAGGCG |
| PVX-DM3-1-F | GGCATGGACGAGCTGTACAAGcccgggATGGCTTCCACTTCCTCTAGCT |
| PVX-DM3-1-R | ATTGCTTTTCTGGAGTTGAAGCGTCCTTCC |
| PVX-DM3-2-F | CTCCAGAAAAGCAATATTTGAGACTGAGTGCCC |
| PVX-DM3-2-R | CTTATCGGCGGTCGACCTAAACTTCTGGCCCAGGCG |
| PVX-DM4-F | GAGCTGTACAAGcccgggATGGCTTCCACTTCCTCTAGCTTCCGATTCTCAGCCAAAAATATTTTCAAGTGCCCATGCACCA |
| PVX-DM4-R | CTTATCGGCGGTCGACCTAAACTTCTGGCCCAGGCG |
| PVX-DM5-1-F | ATGGACGAGCTGTACAAGcccgggATGGCTTCCACTTCCTCTAGCT |
| PVX-DM5-1-R | TTGCACTGTGGGTCTCCGGATTCGTGGAG |
| PVX-DM5-2-F | AGACCCACAGTGCAAGAAACGGGTCGAAACA |
| PVX-DM5-2-R | CTTATCGGCGGTCGACCTAAACTTCTGGCCCAGGCG |
| PVX-DM6-1-F | ATGGACGAGCTGTACAAGcccgggATGGCTTCCACTTCCTCTAGCT |
| PVX-DM6-1-R | TAGTTGCCTTTTCTGGAGGCGGCTGGTGAT |
| PVX-DM6-2-F | CAGAAAAGGCAACTATGTGGAGGAAGGACG |
| PVX-DM6-2-R | CTTATCGGCGGTCGACCTAAACTTCTGGCCCAGGCG |
| PVX-DM7-1-F | ACGAGCTGTACAAGcccgggATGGCTTCCACTTCCTCTAGCTTC |
| PVX-DM7-1-R | TGCAAATTTTTTGGACAGCAGGAATAACACAGTTCA |
| PVX-DM7-2-F | TCCAAAAAATTTGCAACATCACTTCTGGTGTGAC |
| PVX-DM7-2-R | CTTATCGGCGGTCGACCTAAACTTCTGGCCCAGGCG |
| PVX-DM8-F | GACGAGCTGTACAAGcccgggATGGCTTCCACTTCCTCTAGCT |
| PVX-DM8-R | GCTTATCGGCGGTCGACttaTTTTGGACAGCAGGAATAACACAGTTCA |
| **For construction of virus-induced gene silencing vectors** | |
| GFP-EcoRI-F | AGGAATTCTGAGCAAGGGCGAGGAGC |
| GFP-SmaI-R | ATCCCGGGCTGAAGCACTGCACGCCG |
| NbRAR1-EcoRI-F | AGGAATTCTGAGGATGATAACCCTGAAA |
| NbRAR1-SmaI-R | ATCCCGGGTCTCTGGGTTGTGAACCG |
| NbNPR1-EcoRI-F | AGGAATTCTACTGGATATTCTTGACAAAAC |
| NbNPR1-SmaI-R | ATCCCGGGGCAACATTTGTAGTAATTCAAC |
| NbMEK2-EcoRI/F | AGGAATTCCACAGAGATATCAAGCCCTC |
| NbMEK2-SmaI/R | ATCCCGGGCATACAAATGGCGCACATAAGA |
| NbWRKY1-EcoRI-F | AGGAATTCTAAGTCCCATAGTGAACCAC |
| NbWRKY1-SmaI-R | ATCCCGGGCCACTTACGCCTGTTAAACT |
| NbNDR1-EcoRI-F | AGGAATTCATGTCAGACTATGGATCCAA |
| NbNDR1-SmaI-R | ATCCCGGGATTACCTATAGGAAAACTTGTA |
| NbCOI-EcoRI-F | AGGAATTCAGATCTGCCACTTGATAATGG |
| NbCOI/SmaI-R | ATCCCGGGTCTAGAAGGCCTTCATCGGA |
| NbNTF6-EcoRI-F | AGGAATTCCTGATGGAACTCATAAAGAG |
| NbNTF6-SmaI-R | ATCCCGGGATGGAGACTTATCAAGAACGG |
| NbCTR-EcoRI-F | AGGAATTCTCAAATGAGAAATCTGATGTATA |
| NbCTR-SmaI-R | ATCCCGGGTCATGAGAGCAACTGCATGT |
| NbAOX- EcoRI-F | AGGAATATGGCAGTACCAGCAGCAAC |
| NbAOX- SmaI-R | ATCCCGGGCGACAACGTTTGGGTTGGAAG |
| **For qRT-PCR** | |
| qNbActin-97-F | GCAGGAATCCACGAGACTACA |
| qNbActin-97-R | AACCTCCAATCCAGACACTGT |
| RT-NbEF1αF | TATGATTACTGGTACCTCCCC |
| RT-NbEF1αR | ACCTAGCCTTGGAATACTTG |
| RT-NbHIN1F | ATCCTCGGAGTGATTGCATTAG |
| RT-NbHIN1R | TGTTGTTTGTGGTGGACAAATC |
| RT-NbPR1F | TGGTCAATACGGCGAAAAC |
| RT-NbPR1R | GAACCCTAGCACATCC |
| RT-NbPR2F | GCACGACATAACCTTCCACTCTTAG |
| RT-NbPR2R | ACCCTGCTGAATTTGTTCCTTG |
| RT-NbPR3F | GGCATTGGTTCTATTGT |
| RT-NbPR3R | AATTTCTTTCCTACGGGCAGTATCATC |
| RT-NbPR4F | TTTTAAGGAAAGATGCCCAGATGC |
| RT-NbPR4R | GCAGAAGACAACCCTGTAATT |
| RT-NbPR5F | TGTGATGGAGATTTAAG |
| RT-NbPR5R | CAAGTGGTCGGATTTCCATA |
| RT-NbNPR1F | GAAACGCCTATCGGAAACACTG |
| RT-NbNPR1R | AAGCCAATACACTCATTACAGCATC |
| qRT-NbLOX-F | AAAACCTATGCCTCAAGAAC |
| qRT-NbLOX-R | ACTGCTGCATAGGCTTTGG |
| qRT-NbERF1-F | GCTCTTAACGTCGGATGGTC |
| qRT-NbERF1-R | AGCCAAACCCTAGCTCCATT |
| qRT-CYP71D20-F | AAGGTCCACCGCACCATGTCCTTAGAG |
| qRT-CYP71D20-R | AAGAATTCCTTGCCCCTTGAGTACTTGC |
| qRT-NbPTI5-F | CCTCCAAGTTTGAGCTCGGATAGT |
| qRT-NbPTI5-R | CCAAGAAATTCTCCATGCACTCTGTC |
| qRT-NbACRE31-F | AATTCGGCCATCGTGATCTTGGTC |
| qRT-NbACRE31-R | GAGAAACTGGGATTGCCTGAAGGA |
| qRT-NbWRKY7-F | CACAAGGGTACAAACAACACAG |
| qRT-NbWRKY7-R | GGTTGCATTTGGTTCATGTAAG |
| qPCR- NbAOX -F | AAATGGATAGCGAGAGCAGG |
| qPCR- NbAOX -R | CGAACTCATCGGGTCGGTCAAAT |
| **For construction pNmGFPer vectors** | |
| 35s-V1-F | tttggagaggacagggtaccATGGTGAGTACCAGGAGTGGAG |
| 35S-V1-R | attcctgcaggtcgacTTAATTTGATGTAGAATCATAAAAATACAATCGAGCCTGT |
| 35S-V2-F | tttggagaggacagggtaccATGTGTCATTATGCATTAAGTGTTCAAGATTTGC |
| 35S-V2-R | attcctgcaggtcgacTTACACCCCGGAGGAACACC |
| 35S-V3A-F | tttggagaggacagggtaccATGAAACGTGTTGGGCACG |
| 35S-V3A-R | attcctgcaggtcgacTTACCCAGGGCTCCTCCTACG |
| 35S-V3-F | tttggagaggacagggtaccATGGACGGTCAAGATTTGGTGTTACA |
| 35S-V3-R | attcctgcaggtcgacTTAAGCCAAGCTTTTTTTCTTATAATCATTAACATTACTTGTTGG |
| 35S-C1-F | tttggagaggacagggtaccATGGCTTCCACTTCCTCTAGCT |
| 35S-C1-R | attcctgcaggtcgacCTAAACTTCTGGCCCAGGCG |
| 35S-C2-F | tttggagaggacagggtaccATGGCTTCCACTTCCTCTAGCT |
| 35S-C2-R | attcctgcaggtcgacCTAGTAGAATACATCAGTGGGGTCCATATAAT |
